# Nasal pressure swings as the measure of inspiratory effort in spontaneously breathing patients with de novo acute respiratory failure

**DOI:** 10.1186/s13054-022-03938-w

**Published:** 2022-03-24

**Authors:** Roberto Tonelli, Andrea Cortegiani, Alessandro Marchioni, Riccardo Fantini, Luca Tabbì, Ivana Castaniere, Emanuela Biagioni, Stefano Busani, Chiara Nani, Caterina Cerbone, Morgana Vermi, Filippo Gozzi, Giulia Bruzzi, Linda Manicardi, Maria Rosaria Pellegrino, Bianca Beghè, Massimo Girardis, Paolo Pelosi, Cesare Gregoretti, Lorenzo Ball, Enrico Clini

**Affiliations:** 1 Respiratory Diseases Unit, Department of Medical and Surgical Sciences, University Hospital of Modena, University of Modena Reggio Emilia, Modena, Italy; 2 Clinical and Experimental Medicine PhD Program, University of Modena Reggio Emilia, Modena, Italy; 3 Department of Surgical, Oncological and Oral Science (DiChirOnS), University of Palermo, Palermo, Italy; 4 Department of Anesthesia, Intensive Care and Emergency, Policlinico Paolo Giaccone, Palermo, Italy; 5 Intensive Care Unit, University Hospital of Modena, University of Modena Reggio Emilia, Modena, Italy; 6 Department of Surgical Sciences and Integrated Diagnostics, University of Genoa, Genoa, Italy; 7 Anesthesia and Critical Care, IRCCS for Oncology and Neurosciences, San Martino Policlinico Hospital, Genoa, Italy; 8 Fondazione G. Giglio, Cefalù, Italy

**Keywords:** Acute respiratory failure, Non-invasive Mechanical ventilation, Esophageal pressure swings, Nasal pressure swings, Endotracheal intubation, COVID-19, Respiratory monitoring, Inspiratory effort, Self-inflicted lung injury

## Abstract

**Background:**

Excessive inspiratory effort could translate into self-inflicted lung injury, thus worsening clinical outcomes of spontaneously breathing patients with acute respiratory failure (ARF). Although esophageal manometry is a reliable method to estimate the magnitude of inspiratory effort, procedural issues significantly limit its use in daily clinical practice. The aim of this study is to describe the correlation between esophageal pressure swings (Δ*P*_es_) and nasal (Δ*P*_nos_) as a potential measure of inspiratory effort in spontaneously breathing patients with de novo ARF.

**Methods:**

From January 1, 2021, to September 1, 2021, 61 consecutive patients with ARF (83.6% related to COVID-19) admitted to the Respiratory Intensive Care Unit (RICU) of the University Hospital of Modena (Italy) and candidate to escalation of non-invasive respiratory support (NRS) were enrolled. Clinical features and tidal changes in esophageal and nasal pressure were recorded on admission and 24 h after starting NRS. Correlation between Δ*P*_es_ and Δ*P*_nos_ served as primary outcome. The effect of Δ*P*_nos_ measurements on respiratory rate and Δ*P*_es_ was also assessed.

**Results:**

Δ*P*_es_ and Δ*P*_nos_ were strongly correlated at admission (*R*^2^ = 0.88, *p* < 0.001) and 24 h apart (*R*^2^ = 0.94, *p* < 0.001). The nasal plug insertion and the mouth closure required for Δ*P*_nos_ measurement did not result in significant change of respiratory rate and Δ*P*_es_. The correlation between measures at 24 h remained significant even after splitting the study population according to the type of NRS (high-flow nasal cannulas [*R*^2^ = 0.79, *p* < 0.001] or non-invasive ventilation [*R*^2^ = 0.95, *p* < 0.001]).

**Conclusions:**

In a cohort of patients with ARF, nasal pressure swings did not alter respiratory mechanics in the short term and were highly correlated with esophageal pressure swings during spontaneous tidal breathing. Δ*P*_nos_ might warrant further investigation as a measure of inspiratory effort in patients with ARF.

*Trial registration*: NCT03826797. Registered October 2016.

**Supplementary Information:**

The online version contains supplementary material available at 10.1186/s13054-022-03938-w.

## Background

Inspiratory effort producing excessive transpulmonary pressure (P_L_) plays a key role in the progression of lung damage during acute respiratory failure (ARF) of different etiology [1], including severe COVID-19 pneumonia [2]. Negative alveolar pressure, *pendelluft* phenomenon, local overstretch of dependent lung zones and asymmetrical distribution of P_L_ applied to inhomogeneous lung parenchyma are the putative mechanisms of self-inflicted injury (P-SILI) and worse outcomes observed in patients with ARF and breathing spontaneously [3, 4].

Esophageal pressure swings (Δ*P*_es_) mirror the mean P_L_ during non-assisted spontaneous breathing, thus esophageal manometry provides an estimate of the magnitude of inspiratory effort [5] and it is a predictor of non-invasive positive pressure ventilation (NIV) failure [4, 6]. However, esophageal manometry is not easy to implement at the bedside [7], especially in unstable patients with respiratory distress and severe impairment of gas exchange [8, 9]. Notwithstanding, an easy-to-perform respiratory monitoring of patients with ARF would be useful in all patients at risk of P-SILI [8]. In particular, the recent COVID-19 pandemic has increased the number of patients with ARF breathing spontaneously and requiring non-invasive respiratory support (NRS), especially outside the intensive care units (ICUs) [10]. These patients are at high risk of deterioration and could therefore benefit from continuous monitoring of inspiratory effort [11].

Early physiological studies comparing Δ*P*_es_ with nasal (Δ*P*_nos_) and mouth pressure swings, showed no phase difference between pressure waveforms during incremental inspiratory effort [12]. A significant correlation between Δ*P*_es_ and airway pressure swings (Δ*P*_aw_) during an inspiratory effort test, as obtained by an occlusion maneuver, was also observed [13, 14].

The aim of this proof-of-concept physiological study was to describe the correlation between Δ*P*_es_ and Δ*P*_aw_ as captured by Δ*P*_nos_ in a cohort of spontaneously breathing patients with de novo ARF candidate to receive a non-invasive respiratory support (HFNC and NIV). We hypothesized that Δ*P*_es_ and Δ*P*_nos_ were correlated, also during application of different types of NRS.

## Methods

### Study cohort

Patients with ARF admitted to the Respiratory Intensive Care Unit (RICU) at the University Hospital of Modena between January 1^st^, 2021, and September 1^st^, 2021, were prospectively considered eligible for enrollment. This was a pre-planned sub-study of a prospectively registered protocol (ClinicalTrial.gov: ID NCT03826797). The local Ethics Committee (Comitato Etico Area Vasta Emilia Nord) approved the study approval (protocol number 4485/C.E., document 266/16) and written informed consent was obtained from all participants or their relatives, as appropriate.

Inclusion criteria were age > 18 years; presence of ARF with a peripheral oxygen saturation (SpO_2_) < 90% during conventional oxygen therapy with Venturi mask with an inspiratory oxygen fraction (FiO_2_) of 0.5 and candidate to treatment escalation to high flow nasal cannula (HFNC) and consent to receive esophageal and nasal manometry assessment. Exclusion criteria were immediate need for endotracheal intubation; cardiogenic acute pulmonary edema or concomitant hypercapnia (PaCO_2_ > 45 mmHg); previous diagnosis of chronic obstructive pulmonary disease, interstitial lung disease, neuromuscular diseases, anatomical alterations of the nasal tract, or chest wall deformities; use of home long-term oxygen therapy.

### Clinical variables and measurements

**Fig. 1:**
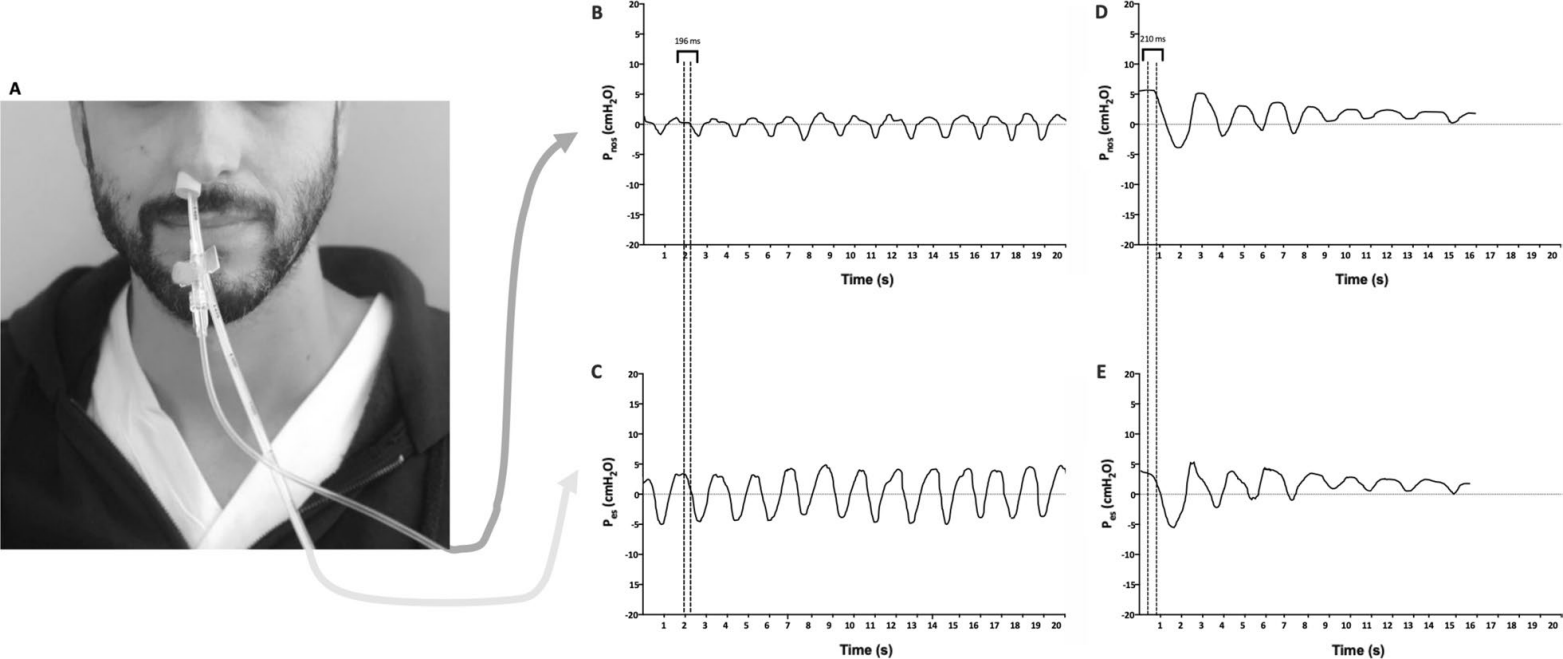
**A** Simultaneous positioning of esophageal catheter for Δ*P*_es_ assessment and nasal plug made of hypoallergenic foam ear plug equipped with a 16 Gauge polyurethane intravenous cannula for Δ*P*_nos_ measurements. The contralateral nostril was kept open. **B**, **C** Simultaneous assessment of Δ*P*_nos_ and Δ*P*_es_ during unsupported spontaneous breathing, showing in phase waveforms with a 196 ms time latency of Δ*P*_nos_ over the onset of inspiratory effort captured by Δ*P*_es_. **D**, **E** Simultaneous assessment of Δ*P*_nos_ and Δ*P*_es_, showing decremental inspiratory effort after NIV placement

Demographics and clinical characteristics, arterial blood gases, the ratio between the partial pressure of oxygen and fraction of inspired oxygen (PaO_2_/FiO_2_ ratio), blood lactate level, and clinical severity as assessed by the Sequential Organ Failure Assessment (SOFA) score, the Acute Physiologic Assessment and Chronic Health Evaluation (APACHE) II, and the Simplified Acute Physiology Score (SAPS) II were collected at the time of RICU admission (T0). At our center, the criteria for being referred to RICU to upgrade to NRS were in accordance with local protocols and included a peripheral oxygen saturation (SpO_2_) < 90% during conventional oxygen therapy with Venturi mask and/or the presence of respiratory rate (RR) > 25 breaths/m(bpm) and/or the presence of subjective respiratory distress. At T0, all patients underwent HFNC initiation whilst esophageal manometry, with a multifunctional nasogastric tube (NutriVent™, SIDAM, Mirandola, Italy) and according to a standardized protocol [4] was provided. The esophageal balloon was connected to a two-channel pressure monitoring system (OptiVent™, SIDAM, Mirandola, Italy) via a 100-cm polyurethane catheter. Δ*P*_es_ and RR were recorded. A custom-made nasal pressure monitoring system was placed in the same nostril as the nasogastric tube, while the contralateral nostril was kept patent. The nasal pressure monitoring system was assembled with one hypoallergenic self-expanding foam ear plug (3 M Company, Saint Paul, Minnesota (MN), USA) with inserted a 16 Gauge polyurethane intravenous cannula. The self-expanding foam plug was placed in the nostril and modeled on the shape of the nasogastric tube to obtain a sealed tight closure of the external surface of the nostril, with HFNC placed only in the patent nostril. The nasal plug was connected to the second channel of the pressure monitoring system to obtain continuous measurement of Δ*P*_nos_. Once the nasal plug was placed and hermetic closure of the nostril was visually checked (T1), Δ*P*_es_ and Δ*P*_nos_, as well as RR, were assessed and recorded simultaneously (see Fig. 1). To evaluate the influence of nasal breathing on the breathing pattern, changes in RR and Δ*P*_es_ after nasal plug placement were assessed. In case of failure of HFNC, patients received a trial of escalation to non-invasive bilevel ventilation (NIV) if deemed indicated by the treating clinician, blinded to the study purposes. The criteria to upgrade to NIV were according to local protocols and included PaO_2_/FiO_2_ ratio < 100 mmHg and/or RR > 25 bpm and/or persistence of respiratory distress and dyspnea despite HFNC set at 60 L/min.

After 24 h (T2), all patients underwent a further simultaneous assessment of Δ*P*_es_ and Δ*P*_nos_, whatever the NRS in use (HFNC or NIV). Measurements at each timepoint were taken while in semi-recumbent position and breathing through the patent nostril with the mouth closed, following a 5-min period with a stable breathing pattern; measures were averaged from 3 subsequent breaths after stabilization. Data were sampled and stored at 100 Hz and processed a posteriori on the pressure monitoring system. The ratio of the Δ*P*_es_ to the Δ*P*_nos_ was computed and its average value was used to compute the estimated Δ*P*_es_ based on the Δ*P*_nos_, according to the formula:

$${\Delta P}_{es, estimated}=k\bullet {\Delta P}_{nos}$$

where k is the mean Δ*P*_es_/Δ*P*_nos_ ratio in the overall cohort at T1, when all patients received HFNC.

### Management of noninvasive respiratory support

Patients did not receive any sedation when using HFNC or NIV. HFNC was delivered with a high flow device (Optiflow^TM^and AIRVO™, Fisher & Paykel Healthcare Ltd, Auckland, New Zealand) through appropriately sized nasal cannulas. Flow delivery was initially set at 60 L/min and oxygen fraction at 50% and temperature at 37 °C then adjusted according to the patient’s tolerance; oxygen fraction was then titrated to target a SpO_2_ ≥ 92%.

NIV was delivered through an appropriately sized total face mask equipped with a dedicated output for probes (DiMax zero™, Dimar, Medolla, Italy) connected to a high-performance ventilator (GE Healthcare Engstrom Carestation™, GE Healthcare, Finland) in non-invasive pressure support mode, ensuring that mask-leak flow was below 20 L/min. The inspiratory trigger was set at 3 L/min and expiratory cycling at 25% of the inspiratory peak flow. Positive end-expiratory pressure (PEEP) was initially set to 8 cmH_2_O and subsequently titrated to target a SpO_2_ > 92% with a delivered FiO_2_ < 0.7, while pressure support was started at 10 cmH_2_O, and then progressively adjusted targeting a tidal volume < 9.5 mL/kg of predicted body weight and a RR < 30 bpm. A heat and moisture exchanger (HME) with antimicrobial properties (Hygrobac, DAR, Mirandola, Italy) was connected between the Y-piece and the mask.

The decision to proceed to endotracheal intubation was taken according to local protocols by the attending staff, blinded to the results of the physiological parameters; criteria included: a) PaO_2_/FiO_2_ ratio unchanged or worsened or below 150 mmHg, b) worsening dyspnea persistence of RR > 35 bpm, c) the need to protect airways due to neurological deterioration or massive secretions, d) hemodynamic instability or major electrocardiographic abnormalities, e) gasping for air, psychomotor agitation requiring sedation, abdominal paradox movements.

### Analysis plan

The correlation between Δ*P*_es_ and Δ*P*_nos_ at any study time and under different NRS was pre-specified as the study goal. The correlation between Δ*P*_es_ and Δ*P*_nos_ at different level of inspiratory effort (above or below the median baseline value of Δ*P*_es_) was also assessed. The distribution of the ratio between Δ*P*_es_ and Δ*P*_nos_ was further described. Normality of data was assessed with visual inspection of quantile–quantile plots, and data are reported as median [interquartile range, IQR], if not stated otherwise. Correlations were sought using Pearson’s *R*, between-groups differences with the Fisher’s and Mann–Whitney tests, as appropriate. Changes in RR and Δ*P*_es_ before and after the insertion of the nasal probe and mouth closing were sought for using the Wilcoxon signed ranks test. A sample size of at least 37 patients would have provided 90% power (1-β) to detect a correlation with *R* > 0.5 at an α level of 0.05. In a sensitivity analysis, we compared the agreement between Δ*P*_es_ measured at T2 and Δ*P*_es, estimated_ based on Δ*P*_nos_ using the Bland–Altman method, to assess whether Δ*P*_nos_ could serve as a surrogate of Δ*P*_es_. We further performed the analysis according to the type of NRS. In another sensitivity analysis, the differences of Δ*P*_es_ and Δ*P*_nos_ in patients that required endotracheal intubation versus those who were still under NIV or HFNC at 3 days from inclusion were assessed. Statistics were performed using *R* (version 4.0.2; *R* Foundation for Statistical Computing, Vienna, Austria). Statistical significance was assumed with two-tailed *p* < 0.05.

## Results

### Patient characteristics

Sixty-one out of 160 consecutive patients admitted to the RICU in the considered time-lapse with de novo ARF and candidates to receive HFNC were enrolled. Of these 51 (83.6%) were diagnosed with COVID-19-related pneumonia while 10 had ARF of different etiology. Reasons for exclusion were: presence of chronic respiratory disease (*N* = 62), unavailability of research staff (*N* = 18), refusal to receive esophageal manometry (*N* = 19). The clinical and physiological characteristics of the study population at the study time points are shown in Table 1.

**Table 1:** General and clinical characteristics of the study population

Variable	At RICU admission (T0)	At study inclusion (T1)	After 24 h (T2)	
Type of respiratory support	HFNC, *N* = 61	HFNC, *N* = 61	HFNC, *N* = 16	NIV, *N* = 45
Diagnosis				
COVID-19, *n* [IQR]	51 [83.6]	–	10 [62.5]	41 [91.1]
ARDS, *n* [IQR]	4 [6.6]	–	1 [6.3]	3 [6.6]
Pneumonia, *n* [IQR]	6 [9.8]	–	5 [31.3]	1 [2.2]
Clinical parameters				
Age, years [IQR]	70 [56–78]	–	69 [45–75]	70 [61–78]
Male sex, [%]	42 [68.9]	–	9 [56.3]	33 [73.3]
Mean arterial pressure, mmHg [IQR]	95 [70–105]	95 [75–105]	90 [70–100]	85 [70–95]
Heart rate, bpm [IQR]	86 [62–110]	86 [62–112]	80 [64–98]	85 [62–112]
SOFA, score, [IQR]	3 [3–3]	–	3 [3–3]	3 [3–3]
SAPSII score, [IQR]	28 [23–33]	–	27 [22–32]	28 [26–33]
APACHEII score, [IQR]	11 [7–14]	–	11 [7–14]	12 [9–15]
Borg scale, value [IQR]	5 [2–8]	–	3 [1–4]	2 [1–4]
Gas exchange				
pH, value [IQR]	7.45 [7.41–7.5]	–	7.44 [7.40–7.49]	7.43 [7.38–7.47]
PaO_2_, mmHg [IQR]	64 [57–72]	–	62 [53–78]	67 [59–75]
PaCO_2_, mmHg [IQR]	34 [31–39]	–	36 [33–41]	37 [34–41]
PaO_2_/FiO_2_, mmHg [IQR]	129 [100–150]	–	147 [118–199]	151 [131–179]
Blood lactate, mmol/L [IQR]	1 [0.4–1.8]	–	0.8 [0.2–1.7]	1 [0.3–1.6]
Respiratory mechanics				
Respiratory rate, *n* [IQR]	25 [24–29]	26 [24–29]	23 [21–25]	22 [20–25]
Δ*P*_es_, cmH_2_O [IQR]	12 [10–7]	12 [10–18]	6.2 [4.8–7]	8 [5.5–11]
Δ*P*_nos_, cmH_2_O [IQR]	–	5.6 [4.2–8.0]	3 [2–3.4]	3.2 [2.3–5.2]
Δ*P*_es_/Δ*P*_nos_, value [IQR]	–	2.2 [2.06–2.49]	2.23 [1.89–2.60]	2.27 [2.15–2.50]

Data are presented as number (*n*) and percentage for dichotomous values or median and interquartile ranges (IQR)) for continuous values

*RICU* respiratory intensive care unit, *COVID-19* Coronavirus 2 disease, *ARDS* acute respiratory distress syndrome, *SOFA* subsequent organ failure assessment, *SAPS* simplified acute physiology score, *APACHE* acute physiology and chronic health evaluation, Δ*P*_es_ esophageal pressure swings, Δ*P*_nos_ nasal pressure swings, *IQR* interquartile range

### Effects of placement of nasal plug on the pattern of breathing

The insertion of the nasal plug did not change the pattern of breathing. The RR before and after insertion of the nasal plug was 25 [24–29] min^−1^ and 26 [24–30] min^−1^, respectively (*p* = 0.12), while the Δ*P*_es_ was 12 [10–17] cmH_2_O and 12 [10–18] cmH_2_O (*p* = 0.29).

### Correlation between Δ*P*_es_ and Δ*P*_nos_

**Fig. 2:**
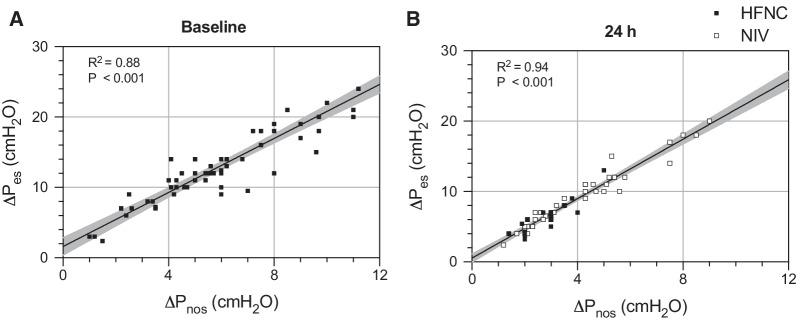
Pearson’s *R* showing correlation between Δ*P*_es_ and Δ*P*_nos_ at baseline (**A**), when all patients were assisted with HFNC (*R*^2^ = 0.88, *p* < 0.001), and at 24 h (**B**, *R*^2^ = 0.94, *p* < 0.001), with most patients receiving NIV. At both time points Δ*P*_es_ and Δ*P*_nos_ showed strong correlation

At T1, median Δ*P*_es_ and Δ*P*_nos_ were 12 [10–17] cmH_2_O and 5.6 [4.2–8.0] cmH_2_O, while at T2 were 7 [5–11] cmH_2_O and 3.0 [2.1–4.7] cmH_2_O, respectively. Patients with COVID-19 presented lower values of Δ*P*_es_ and Δ*P*_nos_ at T1 but not at T2 (Additional file 1: eTable 1). Figure 2 shows the correlation between Δ*P*_es_ and Δ*P*_nos_ at T1 (Panel A), with all patients receiving HFNC (*R*^2^ = 0.88, *p* < 0.001), and at T2 (Panel B, *R*^2^ = 0.94, *p* < 0.001), with 16 (26.2%) and 45 (73.8%) under HFNC and NIV, respectively. The correlation at T2 remained significant when analyzing separately patients receiving HFNC (*R*^2^ = 0.79, *p* < 0.001) or NIV (*R*^2^ = 0.95, *p* < 0.001) (Additional file 1: eFigure 1).

### Δ*P*_es_ to Δ*P*_nos_ ratio

**Fig. 3:**
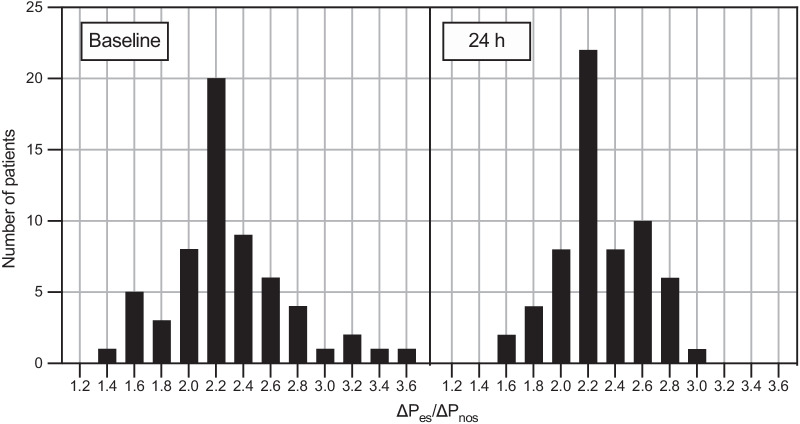
Histogram bars illustrating the distribution of Δ*P*_es_/Δ*P*_nos_ ratio at baseline and at 24 h. The ratio was not different between baseline and 24 h (*p* = 0.41)

The distribution of the Δ*P*_es_/Δ*P*_nos_ ratio is illustrated in Fig. 3 and was similar at T1 and T2 (2.20 [2.06–2.47] and 2.27 [2.11–2.50], *p* = 0.41). The Δ*P*_es_/Δ*P*_nos_ ratio at T2 was similar in patients receiving HFNC versus NIV (2.23 [1.89–2.60] and 2.27 [2.15–2.50], respectively, *p* = 0.63). Moreover, the Δ*P*_es_/Δ*P*_nos_ ratio at T1 was similar in patients with low versus high respiratory drive, defined as Δ*P*_es_ > 12 cmH_2_O, (2.19 [2.06–2.50] and 2.20 [2.06–2.38], *p* = 0.67).

### Sensitivity analyses

The mean Δ*P*_es_/Δ*P*_nos_ ratio at T1 was 2.27 (standard deviation 0.44), and this value was used as multiplication factor to compute Δ*P*_es, estimated_ from Δ*P*_nos_. Bland–Altman method at T2 showed a bias of 0.1 cmH_2_O and 95% limits of agreement, LoA, from -2.0 to 2.1 cmH_2_O (95.1% of measurements within LoA, Additional file 1: eFigure 2). Among patients receiving HFNC bias was 0.1 cmH_2_O and 95% LoA were from -2.1 to 2.3 cmH_2_O, while in patients receiving NIV bias was 0.0 and 95% LoA from -2.0 to 2.0 cmH_2_O.

Characteristics of patients who were intubated versus those still on NRS at day 3 are displayed in Additional file 1: eTable 2: at inclusion, Δ*P*_es_ was 14.0 [10–18.0] and 12.0 [10.0–16.0] cmH_2_O (*p* = 0.53), while Δ*P*_nos_ was 6.5 [4.3–8.4] and 5.6 [4.3–7.5] cmH_2_O (*p* = 0.76), respectively. At 24 h, patients who were further intubated had both higher Δ*P*_es_ (15.0 [12.0–18.0] versus 7.0 [5.0–8.0] cmH_2_O, *p* < 0.001) and Δ*P*_nos_ (7.5 [5.5–7.9] versus 3 [2.1–3.5] cmH_2_O, *p* < 0.001) as compared with those still under NRS.

## Discussion

The main findings of this study are that, in a population of patients with prevalently COVID-19-induced ARF, Δ*P*_nos_ measured with closed mouth was highly correlated with Δ*P*_es_ during non-assisted and assisted spontaneous breathing. The correlation between these two physiological variables showed persistence over time and low inter-patient variability regardless the application of HFNC or NIV. The assessment of Δ*P*_nos_ did not affect inspiratory effort and respiratory rate during non-assisted spontaneous breathing. Moreover, Δ*P*_nos_ and Δ*P*_es_ after 24 h from NRS start resulted significantly higher in those patients that were subsequently intubated.

To our knowledge, this is the first study assessing the correlation between tidal changes of esophageal and nasal pressure in patients with ARF during spontaneous breathing. Previous studies already reported that *P*_es_ could be estimated by nasal pressure during a sniff test [12]. However, sniff represents a ballistic maneuver characterized by an acute increase in lung volume associated with a distortion of the chest wall, far from an isometric contraction [15]. Moreover, during volitional maximal inspiratory effort, the nasal valve of the patent nostril collapses, thus behaving as a Starling resistor. The pressure measured beyond the collapsed segment was found to closely reflect esophageal pressure with an average ratio *P*_es_/*P*_nos_ of 1.05 during maximal sniff and of 1.09 during submaximal sniff, being *P*_nos_ always less than *P*_es_. During tidal spontaneous breathing, instead, the posterior nasal valve remains open. When *P*_nos_ and *P*_es_ are measured during spontaneous breathing without the collapse of the posterior nasal valve, simultaneous pressure waveforms did not show phase difference, though the pressure ratio increased [12]. Based on these assumptions, *P*_nos_ swing is likely to mirror variation of *P*_es_ during spontaneous breathing. Indeed, our study confirmed that Δ*P*_nos_ was highly correlated with Δ*P*_es_ with a narrow range of ratio between the two values, irrespective of the strength of the inspiratory effort.

The distribution of Δ*P*_es_/Δ*P*_nos_ ratio across our patient cohort was relatively consistent showing low inter-patient variability over time and under different type of support (Fig. 3). Given that Δ*P*_nos_ reflects the P_aw_ variation during tidal breathing, a potential interference in Δ*P*_nos_ and Δ*P*_es_/Δ*P*_nos_ ratio assessment following the application of positive inspiratory pressure could have been hypothesized. However, our results suggested that these measurements are not affected by the onset of positive pressure support ventilation. This might be because of the nasal plug that makes the nostril cavity an isolated anatomical structure not influenced by external pressure. This mechanism could explain why the Δ*P*_es_/Δ*P*_nos_ ratio remains constant over time regardless the application of NIV. If confirmed in heterogeneous cohorts of patients with ARF, these preliminary data might suggest monitoring Δ*P*_nos_ as a non-invasive and easy-to-perform surrogate measure of Δ*P*_es_ to monitor the patient’s inspiratory effort during both assisted and not assisted spontaneous breathing.

The magnitude of inspiratory effort as assessed by esophageal manometry was not very high in our population. This might be because most of the enrolled patients were diagnosed with COVID-19 who displayed lower baseline values of Δ*P*_es_ and Δ*P*_nos_. A recently published matched study by our group comparing patients with moderate to severe ARF [16], showed a relatively low activation of respiratory drive in COVID-19 patients during the early phase as compared with classical ARDS population. Despite the limited inspiratory effort observed during tidal breathing, patients in the present study still showed Δ*P*_es_ levels above physiological ranges [17], thus reinforcing the suggestion of monitoring respiratory effort in patients with ARF and prompted to assisted breathing. Further, the implementation of NIV decreased the inspiratory effort which translated into a reduction of both ∆*P*_es_ and ∆*P*_nos_ at 24 h. According to a work from our group [4], a reduction of ∆*P*_es_ lower than 10 cmH_2_O after 2 h of NIV was found to be associated with a higher risk of NIV failure. Whether a threshold of ∆*P*_nos_ reduction following NIV could be determined as a predictor of intubation should be investigated in larger cohorts.

Although our study reported high correlation between Δ*P*_es_ and Δ*P*_nos_, this technique may suffer from several physiological limitations that deserve discussion. First, previous studies regarding Sniff Nasal Inspiratory Pressure (SNIP) test showed that the transmission of pressure changes from the alveoli to the upper airways is altered in case of airflow limitation [18]. Moreover, SNIP was found to underestimate sniff Δ*P*_es_ on average by 14% in patients with acute asthma and by 19% in patients with stable COPD [19, 20]. Despite Δ*P*_nos_ and SNIP exhibit different physiological behaviors, dynamic hyperinflation may affect Δ*P*_es_/Δ*P*_nos_ ratio also during spontaneous breathing. As we have excluded patients with hypercapnic respiratory failure and chronic obstructive respiratory disease, the present results should not be automatically extended to patients affected by significant dynamic intrinsic positive end expiratory pressure. Second, the measurement of Δ*P*_nos_ during spontaneous breathing may be affected by the collapse of the posterior nasal valve induced by exaggerated respiratory drive [15, 21]. In this circumstance, tidal inspiratory breathing may become similar to an inspiratory effort against a closed airway, thus amplifying the pressure variation captured in the nostril. In this line, a device able to maintain the posterior nasal valve open could be useful to obtain reliable value of Δ*P*_nos_. Third, all the measurements were performed with patients asked to keep the mouth closed for the entire evaluation time. This task, however, might be difficult to accomplish in some clinical conditions (e.g., elevated respiratory drive, intense shortness of breath, lack of collaboration), and during severe nasal congestion or anatomical alterations of the nostrils. Moreover, although we did not measure the flow change induced by the Δ*P*_nos_ equipment, it cannot be excluded that the insertion of the nasal plug would have modified the flow (and thus the positive pressure) delivered through HFNC. In this line, there is evidence that closing a nostril can modify the final flow delivered through HFNC, thus reducing the degree of positive pressure generated [22]. However, Bräunlich et al. [23] demonstrated that flow did not change by occluding one of the prongs of the nasal cannula. They also found that delivering pressure at 40 L/m slightly changed end positive airway pressure in this condition even leaving the contralateral nostril open. In addition, given that each measurement lasted for very limited time (5 min) and that we did not find significant changes in the breathing pattern (RR and Δ*P*_es_) after placing the *P*_nos_ equipment, we feel that the plug insertion did not significantly affect the impact provided by HFNC on patients’ respiratory drive. Also, our study population mainly included patients with COVID-19-related ARF, much reducing the generalizability of results. Finally, the clinical evidence provided by our investigation should be taken with caution as the study was not sufficiently empowered. In this line we believe that a different study design with a pre-calculated sample size would be needed to find a reliable threshold of ∆*P*_es_ and ∆*P*_nos_ able to predict the risk for intubation.

## Conclusions

With this proof-of-concept physiological study we have showed that nasal pressure swing during spontaneous tidal breathing was highly correlated with esophageal pressure swing in patients with ARF. The ratio between these variables showed persistence over time and low inter-patient variability regardless the application of NRS. Should data be confirmed on larger studies and in heterogeneous populations with ARF, noninvasive and easy-to-use measurement of Δ*P*_nos_ could be extended in different settings of care, thus implementing the respiratory effort monitoring of patients with ARF at impending risk of deterioration.

## Supplementary Information

**Additional file 1: eTable 1.** Esophageal and nasal pressure swings according to acute respiratory failure etiology. Data are presented as median and interquartile ranges (IQR). **eFigure 1.** Pearson’s R showing correlations between Δ*P*_es_ and Δ*P*_nos_ at 24 hours after splitting the study population according to the NRS received. **eFigure 2.** Bland-Altman analysis assessing the agreement between Δ***P***_es_ measured with esophageal manometry and estimated based on Δ*P*_nos_ (Δ*P*_es, estimated_) and computed as k·Δ*P*_nos_, where k is the average ratio of Δ*P*_es_ to Δ*P*_nos_ measured at baseline. At T2 Bland-Altman methods showed a bias of 0.1 cmH_2_O and 95% limits of agreement, LoA, from −2.0 to 2.1 cmH_2_O (95.1% of measurements within LoA). Among patients receiving HFNC bias was 0.1 cmH_2_O and 95% LoA were from −2.1 to 2.3 cmH_2_O, while in patients receiving NIV bias was 0.0 and 95% LoA from −2.0 to 2.0 cmH_2_O. **eTable 2.** General and clinical characteristics of the study population according to respiratory support at day 3. Data are presented as number (n) and percentage for dichotomous values or median and interquartile ranges (IQR) for continuous values.

## Data Availability

Data are available at the Respiratory Disease Unit of the University Hospital of Modena, Italy.

## Abbreviations

ARF: Acute respiratory failure
NRS: Non-invasive respiratory support
NIV: Non-invasive mechanical ventilation
HFNC: High flow nasal cannula
P_L_: Transpulmonary pressure
Δ*P*_es_: Esophageal pressure swings
Δ*P*_nos_: Nasal pressure swings
SARS-CoV-2: Severe Acute Respiratory Syndrome Coronavirus-2
COVID-19: SARS-CoV-2-induced Disease
RICU: Respiratory Intensive Care Unit
RR: Respiratory rate
SOFA: Sequential Organ Failure Assessment
APACHE II: Acute Physiologic Assessment and Chronic Health Evaluation
SAPS II: Simplified Acute Physiology Score
PEEP: Positive end-expiratory pressure
PS: Pressure support
PT-PCR: Real-time polymerase chain reaction
BPM: Breaths per minute
IQR: Interquartile range
FiO_2_: Fraction of oxygen
ETI: Endotracheal intubation
ROC: Receiver operating characteristic
SNIP: Sniff Nasal Inspiratory Pressure
Δ*P*_aw_: Airway pressure swings
