# Supplementary material for: Nasal pressure swings as the measure of inspiratory effort in spontaneously breathing patients with de novo acute respiratory failure
Source: Crit Care. 2022 Mar 24;26:70. doi: 10.1186/s13054-022-03938-w (PMC8943795; doi:10.1186/s13054-022-03938-w)
Supplement: Supplementary file 1 — Additional file 1: eTable 1. Esophageal and nasal pressure swings according to acute respiratory failure etiology. Data are presented as median and interquartile ranges (IQR). eFigure 1. Pearson’s R showing correlations between ΔPes and ΔPnos at 24 hours after splitting the study population according to the NRS received. eFigure 2. Bland-Altman analysis assessing the agreement between ΔPes measured with esophageal manometry and estimated based on ΔPnos (ΔPes, estimated) and computed as k·ΔPnos, where k is the average ratio of ΔPes to ΔPnos measured at baseline. At T2 Bland-Altman methods showed a bias of 0.1 cmH2O and 95% limits of agreement, LoA, from −2.0 to 2.1 cmH2O (95.1% of measurements within LoA). Among patients receiving HFNC bias was 0.1 cmH2O and 95% LoA were from −2.1 to 2.3 cmH2O, while in patients receiving NIV bias was 0.0 and 95% LoA from −2.0 to 2.0 cmH2O. eTable 2. General and clinical characteristics of the study population according to respiratory support at day 3. Data are presented as number (n) and percentage for dichotomous values or median and interquartile ranges (IQR) for continuous values. [file 13054_2022_3938_MOESM1_ESM.docx]

**Supplementary materials**

**eTable 1.**

| Variable | COVID-19  (n=51) | Other than COVID-19 (n=10) | p value |
| --- | --- | --- | --- |
| *T1 ΔP_es_, cmH_2_O [IQR]* | 12 [9.4 – 14.3] | 16 [10 – 28] | 0.01 |
| *T1 ΔP_nos_, cmH_2_O [IQR]* | 5.6 [4.2 – 7.6] | 6.7 [4 – 12.5] | 0.04 |
| *T2 ΔP_es_, cmH_2_O [IQR]* | 6.8 [5 – 10] | 8.5 [7 – 12.3] | 0.3 |
| *T2 ΔP_nos_, cmH_2_O [IQR]* | 3 [2.1 – 4.6] | 3.9 [2.7 – 5.1] | 0.7 |

Esophageal and nasal pressure swings according to acute respiratory failure etiology. Data are presented as median and interquartile ranges (IQR).

*COVID-19 = Coronavirus 2 disease; ΔP_es_ = esophageal pressure swings; ΔP_nos_ = nasal pressure swings.*

**eFigure 1.**

Pearson’s R showing correlations between ΔP_es_ and ΔP_nos_ at 24 hours after splitting the study population according to the NRS received.

**eFigure 2.**

Bland-Altman analysis assessing the agreement between ΔP_es_ measured with esophageal manometry and estimated based on ΔP_nos_ (ΔP_es,estimated_) and computed as k· ΔP_nos_, where k is the average ratio of ΔP_es_ to ΔP_nos_ measured at baseline. At T2 Bland-Altman methods showed a bias of 0.1 cmH_2_O and 95% limits of agreement, LoA, from -2.0 to 2.1 cmH_2_O (95.1% of measurements within LoA). Among patients receiving HFNC bias was 0.1 cmH_2_O and 95% LoA were from -2.1 to 2.3 cmH_2_O, while in patients receiving NIV bias was 0.0 and 95% LoA from -2.0 to 2.0 cmH_2_O.

**Table e2.**

General and clinical characteristics of the study population according to respiratory support at day 3. Data are presented as number (n) and percentage for dichotomous values or median and interquartile ranges (IQR)) for continuous values.

| Variable | Still on NRS at day 3  (n=51) | Intubated at day 3 (n=10) | p value |
| --- | --- | --- | --- |
| *Age, years [IQR]* | 70 [60 – 75] | 73 [59 – 78] | 0.8 |
| *Male sex, n [%]* | 34 [67] | 9 [90] | 0.2 |
| *SOFA, score, [IQR]* | 3 [3 – 3] | 3 [3 – 3] | 0.4 |
| *SAPSII score, [IQR]* | 27 [24 – 33] | 28 [25 – 33] | 0.5 |
| *APACHEII score, [IQR]* | 11 [9 – 14] | 11 [9 – 15] | 0.7 |
| *T0 PaO_2_/FiO_2_, mmHg [IQR]* | 133 [116 – 156] | 110 [103 – 125] | 0.1 |
| *T0 respiratory rate, bpm [IQR]* | 26 [24 – 29] | 27 [24 – 31] | 0.5 |
| *T1 ΔP_es_, cmH_2_O [IQR]* | 12 [10 – 16] | 14 [10 – 18] | 0.5 |
| *T1 ΔP_nos_, cmH_2_O [IQR]* | 5.6 [4.3 – 7.5] | 6.5 [4.3 – 8.4] | 0.8 |
| *HFNC, n [%]* | 16 [31.4] | 0,0% | --- |
| *NIV, n [%]* | 35 [68.6] | 0,0% | --- |
| *T2 PaO_2_/FiO_2_, mmHg [IQR]* | 153 [131 – 190] | 128 [86 – 152] | 0.03 |
| *T2 respiratory rate, bpm [IQR]* | 21 [20 – 24] | 28 [25 – 30] | 0.001 |
| *T2 ΔP_es_, cmH_2_O [IQR]* | 7 [5 – 8] | 15 [12 – 18] | <0.001 |
| *T2 ΔP_nos_, cmH_2_O [IQR]* | 3 [2.1 – 3.5] | 7 [5.5 – 7.9] | <0.001 |

*NRS = non-invasive respiratory support; SOFA = subsequent organ failure assessment; SAPS = simplified acute physiology score; APACHE = acute physiology and chronic health evaluation; ΔP_es_ = esophageal pressure swings; ΔP_nos_ = nasal pressure swings; HFNC = high flow nasal cannula; NIV = Non-invasive mechanical ventilation; IQR = interquartile range*
